# Exosomes derived from mature dendritic cells increase endothelial inflammation and atherosclerosis *via* membrane TNF‐α mediated NF‐κB pathway

**DOI:** 10.1111/jcmm.12923

**Published:** 2016-08-12

**Authors:** Wei Gao, Haibo Liu, Jie Yuan, Chaoneng Wu, Dong Huang, Yuanji Ma, Jianbing Zhu, Leilei Ma, Junjie Guo, Hongtao Shi, Yunzeng Zou, Junbo Ge

**Affiliations:** ^1^ Shanghai Institute of Cardiovascular DiseasesZhongshan HospitalFudan UniversityShanghaiChina; ^2^ Institute of Biomedical ScienceFudan UniversityShanghaiChina

**Keywords:** dendritic cells, exosomes, TNF‐α, endothelial inflammation, atherosclerosis

## Abstract

Whether dendritic cell (DC) derived exosomes play a role in the progression of endothelial inflammation and atherosclerosis remains unclear. Using a transwell system and exosome release inhibitor GW4869, we demonstrated that mature DCs contributed to endothelial inflammation and exosomes were involved in the process. To further confirm this finding, we isolated exosomes from bone marrow dendritic cell (BMDC) culture medium (named DC‐exos) and stimulated human umbilical vein endothelial cell (HUVEC) with these DC‐exos. We observed that mature DC‐exos increased HUVEC inflammation through NF‐κB pathway in a manner similar to that of lipopolysaccharide. After a protein array analysis of exosomes, we identified and confirmed tumour necrosis factor (TNF)‐α on exosome membrane being the trigger of NF‐κB pathway in HUVECs. We then performed an *in vivo* study and found that the aorta endothelial of mice could uptake intravenously injected exosomes and was activated by these exosomes. After a period of 12 weeks of mature DC‐exos injection into ApoE−/− mice, the atherosclerotic lesions significantly increased. Our study demonstrates that mature DCs derived exosomes increase endothelial inflammation and atherosclerosis *via* membrane TNF‐α mediated NF‐κB pathway. This finding extends our knowledge on how DCs affect inflammation and provides a potential method to prevent endothelial inflammation and atherosclerosis.

## Introduction

Atherosclerotic vascular disease remains the first cause of death and morbidity. It is now well‐established that atherosclerosis is a chronic inflammatory disease of the vessel wall. The atherosclerotic plaque is characterized by an accumulation of lipids in the artery wall, together with the infiltration of immunocytes and the formation of a fibrous cap 1. Besides macrophages, dendritic cells (DCs) can be also found within atherosclerotic lesions and contribute to atherosclerosis 2. In healthy arteries, only small numbers of immature DCs are detected. During atherosclerosis development, the immature DCs go mature and the numbers dramatically increase. They participate in all stages of atherosclerosis from fatty streaks to mature lesions through contributing to accumulation of lipids, antigen presentation, cytokine production, chemokine production and tertiary lymphoid organ formation 3. The first step of atherosclerosis is activation of endothelial cells, which elaborate adhesion molecules such as vascular cell adhesion molecule‐1 (VCAM‐1), intercellular adhesion molecule 1 (ICAM‐1) and E‐selectin. Helped with these adhesion molecules, monocytes and probably neutrophils attach to the endothelium at first loosely then firmly and move into the sub‐endothelial space, where they go transformation and move the atherosclerotic process to next step. Endothelial cells can be activated by various factors, among which a number of pro‐inflammatory cytokines are produced by mature DCs, including tumour necrosis factor (TNF), interleukin (IL)‐6 and IL‐12 3, 4.

Traditionally, the transfer of information between cells can be achieved by direct contact and cytokines. In the past decade, however, extracellular vesicles have been recognized as potent vehicles of intercellular communication because of their capacity of transferring proteins, lipids and nucleic acids, thereby influencing various physiological and pathological functions of both recipient and parent cells. Exosomes are a subset of extracellular vesicles released by almost all kinds of cell types. It has been reported that exosomes contain a variety of biological components, including membrane proteins, lipids, RNA and even DNA 5. Upon being released from cells, exosomes can distribute in biological fluids and be up‐taken by the same or a different type of cells, which then interact with exosomes and present biological functions 6. Previous studies showed that DC‐derived exosomes (DC‐exos) can exert biological effects *via* membrane proteins or inner contained miRNAs 7, 8, 9. Here, in the present study, we aimed at investigating whether DC‐exos are involved in endothelial inflammation and atherosclerosis.

We firstly demonstrated that mature DCs could activate human umbilical vein endothelial cells (HUVECs) and DC‐exos were involved in this process. We then investigated the mechanism of DC‐exos activating endothelial cells. Our findings revealed a rapid activation of NF‐κB pathway in HUVECs. NF‐κB signalling is a widely investigated pathway, whose activation in HUVECs induces transcription of adhesion molecules such as VCAM‐1, ICAM‐1 and E‐selectin. The canonical pathway of activating NF‐κB signalling is through the ligation of TNF receptor 1 and soluble or membrane‐bound TNF‐α. We then demonstrated that TNF‐α exists in the membrane of mature DC‐exos. When we down‐regulated or neutralized TNF‐α in mature DC‐exos, the activation of NF‐κB pathway and HUVECs were attenuated. Finally, we intravenously injected PKH67‐labelled DC‐exos into mice and observed that these exosomes could be up‐taken by aortic endothelial cells. As a result, the endothelial inflammation increased and, in ApoE−/− mice, the atherosclerosis progressed. Our results showed a novel mechanism of DCs involvement in atherosclerosis.

## Materials and methods

### Dendritic cells culture and transfection

As described previously, bone marrow dendritic cells (BMDCs) were obtained from C57BL/6 mice 10. To eliminate the interference of exosomes from foetal bovine serum, we used a non‐serum medium, X‐VIVO 15 (Lonza, Basel, Swiss), to culture BMDCs 11. Briefly, the mice were sacrificed by cervical dislocation and bone marrow progenitors were washed out from long bones (thigh‐bone and shin bone) and cultured in medium containing 10 ng/ml granulocyte‐macrophage colony‐stimulating factor and 1 ng/ml IL‐4 (PeproTech, New Jersey, USA). Non‐adherent cells were gently washed out at 48 hrs. The remaining clusters, which were loosely adherent to Petri dish, were cultured and the medium was changed every other day. On day 7, the cells were collected for treatment and they were treated with different protocols depending on different studies subsequently conducted.

For exosome isolation, DCs were treated with lipopolysaccharide (LPS, 5 μg/ml) or PBS for 24 hrs and afterwards DCs were washed twice with PBS and replaced with fresh medium. After another 48 hrs of continuous culturing, the culture medium was collected for exosome isolation according to the manufacturer's protocol. A brief protocol can be found in ‘Exosomes isolation, analysis, labelling and neutralization’ part.

For transfection study, DCs were transfected with siRNA TNF‐α for 24 hrs and treated with LPS or PBS for another 12 hrs. The DCs were then washed twice with PBS and replaced with fresh medium. After another 36 hrs of continuous culturing, the culture medium was collected for exosome isolation. A transfection kit riboFECT^™^ CP (Ribobio, Guangzhou, China) was used. SiRNA TNF‐α was also purchased from Ribobio and the transfected concentration was 50 nM.

### HUVECs culture and intervention

Primary HUVECs were purchased from AllCells (Shanghai, China) and cultured with a special medium ECM (ScienCell, San Diego, CA, USA). A total of two batches were obtained and studied. The 8–10 generations were used for our study and cells were collected for mRNA or protein detection after treatment with exosomes for 6 or 24 hrs respectively. To make our data more reliable, three independent experiments were performed. The first one was performed with one HUVEC batch and the other two were performed with another HUVEC batch.

### Co‐culture of DCs and HUVECs

To clarify whether exosomes are involved in the process of endothelial inflammation induced by DCs, we co‐cultured DCs with HUVECs in a Transwell‐6 system with a 0.4‐μm porous membrane (Corning, Corning, NY, USA) to prevent both the transfer of vesicles larger than exosomes and direct cell contact. The HUVECs were planted in the lower wells and grown for an appropriate period of time. The DCs were then planted in the upper wells. GW4869 (Sigma‐Aldrich, California, USA) was used at a concentration of 10 μM to reduce the release of exosome from DCs 12. Before DCs were co‐cultured with HUVECs, they were stimulated with GW4869 for 8 hrs.

### Exosomes isolation, analysis, labelling and neutralization

Serum‐free conditioned medium was collected at the indicated time‐points after cell treatment. Exosomes were precipitated using exosome precipitation solution (Exo‐Quick; System Bioscience, Missouri, USA) following the manufacturer's instructions with some modifications 13, 14. Briefly, the culture medium was harvested at 3000 × g for 15 min. and then underwent a centrifugation at 10,000 × g for 30 min. to eliminate cell debris. The obtained supernatant was then filtrated with 0.22 μm filter to further eliminate cell debris and large particles. Exo‐Quick was added to the medium at a ratio of 1:5 and the mixed solution was placed at 4°C over night. We then centrifuged the solution at 1500 × g for 30 min., resuspended the exosome pellet with PBS and stored them at −80°C for subsequent studies.

The ultrastructure and size distribution of exosomes were analysed by transmission electron microscopy and Nanosight (Malvern, Malvern, UK) respectively. Protein markers, CD63, Alix, TSG101 and Calnexin were determined by immunoblotting.

For up‐take studies, purified exosomes were labelled with a PKH67 (green) kit (Sigma‐Aldrich) according to protocols previously reported 15. Briefly, exosomes diluted in PBS were added to 0.5 ml Diluent C. In parallel, 4 μl PKH26/PKH67 dye was added to 0.5 ml Diluent C and incubated with the exosome solution for 4 min. at room temperature. To bind excess dye, 2 ml 0.5% bovine serum albumin/PBS was added. The labelled exosomes were washed at 100,000 × g for 1 hr, and the exosome pellet was suspended with PBS and used for uptake experiments. After cells were fixed, they were visualized with a confocal fluorescence microscope or immunofluorescent microscopy.

For neutralization study, TNF‐α antibody (BD Pharmingen, San Diego, CA, USA) was used to neutralize TNF‐α on exosomal membrane at 37°C for 6 hrs. The incubated concentration was 100 μg/ml.

### Real‐time q‐PCR

Total RNA was extracted using TRIzol reagent (Sangon, Shanghai, China) from homogenized tissues or cells. ReverTra Ace qPCR RT Kit (Toyobo, Osaka, Japan) was used to generate cDNA from mRNA and SYBR Premix Ex Taq (Takara, Kyoto, Japan) was used for real‐time qPCR with the ABI 7500 Real‐time PCR system following the manufacturer's instructions. Primers for mouse VCAM‐1, ICAM‐1, E‐selectin, IL‐1, IL‐6, TNF‐α and human VCAM‐1, ICAM‐1, E‐selectin are listed in Table S1.

### Western blot analysis

Isolated exosome pellet was lysed in RIPA (Radio‐Immunoprecipitation Assay) buffer supplemented with complete protease inhibitor cocktail tablets (Roche, Basel, Swiss). Cultured HUVECs were harvested and lysed in RIPA buffer supplemented with complete protease inhibitor cocktail tablets. Cell debris was removed by centrifugation at 13,400 g for 20 min. Then the lysates of exosomes or cells were separated by SDS‐PAGE gels, transferred to PVDF membranes (Bio‐Rad, California, USA), and incubated with the relevant antibodies as indicated. Quantity One software was used to calculate the abundance of protein. Antibody against CD63 was purchased from Santa Cruz Biotechnology (California, USA), p‐p105 and Alix were purchased from Cell Signaling Technology (Massachusetts, USA) and VCAM‐1, TSG101 and Calnexin were purchased from Abcam (Cambridge, UK).

### Flow cytometry

Cells were harvested and counterstained immunophenotypically using anti‐CD83, anti‐CD80, anti‐CD86, anti‐CD40 and anti‐CD11c (BD Pharmingen).

### Elisa and protein chip analysis

The BMDCs culture supernatant was analysed for IL‐1, IL‐6, IL‐10, IL‐12p70 and TNF‐α with ELISA kits according to manufacturer's protocol (R&D Systems, Minneapolis, MN, USA). For protein chip analysis, exosomes were harvested and lysed or suspended with PBS. After an adjustment of concentration, the solution was analysed with a mouse inflammation protein array (RayBiotech, Georgia, USA) according to manufacturer's protocol.

### Exosomes uptake and characterization of atherosclerotic lesions in mice

For exosomes uptake study *in vitro*, PKH67‐labelled exosomes were co‐cultured with HUVECs for indicated time. Then HUVECs were fixed and stained with phalloidin (Life Technologies, California, USA) and DAPI (Life Technologies). The images were obtained by an immunofluorescent microscope.

For exosomes uptake study *in vivo*, mice were fixed with a fixation device and PKH67‐labelled exosomes were intravenously injected to mice. The fixation device was purchased from GlobalBio (Beijing, China) and it can make the mice stable while they are awake. After 24 hrs, mice were sacrificed by cervical dislocation and aortas were carefully excised from mice and embedded in OCT compound and frozen at −80°C. Serial cryostat sections of the aortic arch were prepared using a Lab‐Tek tissue processor (Leica, Solms, Germany).The aortic sections were stained with CD31 (Abcam) and DAPI. Images were acquired on an upright Leica SP8 confocal microscope.

To induce endothelial inflammation in mice, 20 μg exosomes in 100 μl PBS or 100 μl PBS were injected to 8‐week‐old male C57BL/6 mice. After 24 hrs or 3 days, mice were killed by cervical dislocation and aortas were carefully excised. The total RNA was extracted for detection of VCAM‐1, ICAM‐1 and E‐Selectin.

For characterization of atherosclerotic lesions, 20 μg exosomes in 100 μl PBS or 100 μl PBS were injected weekly to 8‐week‐old male ApoE−/− mice for a period of 12 weeks. Meanwhile, these mice were fed with a high fat diet. The mice were then killed by cervical dislocation and the basal portion of the heart and aortic roots were embedded in OCT compound and frozen at −80°C. Serial cryostat sections were prepared and Oil red O staining was performed. The lesion areas were determined with ImageJ (National Institutes of Health, Maryland, USA).

### Statistical analysis

For quantification analysis, at least three independent experiments were performed. The results were shown as mean value ± S.D. Student's *t*‐test was used to determine statistical significance between groups. For comparison of three or more groups, one‐way anova test was applied. A *P* < 0.05 was considered statistically significant.

## Results

### Mature DCs contribute to endothelial inflammation and exosomes are involved in the process

**Figure 1:**
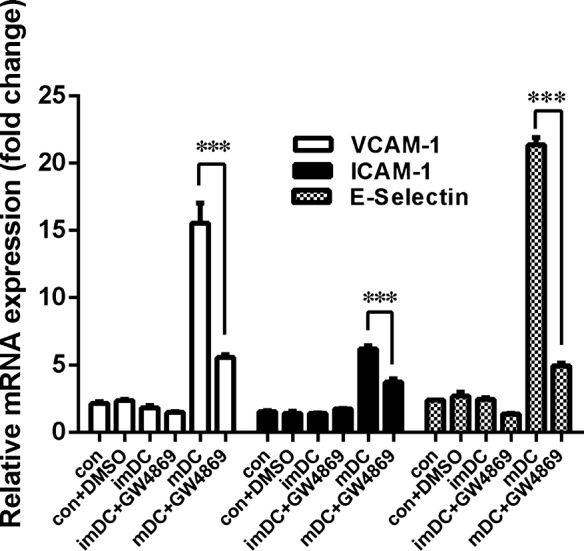
The co‐culture of DCs and HUVECs in a transwell system. BMDCs and HUVECs were co‐cultured in a transwell system and GW4869, a well‐known inhibitor of exosome secretion, is used at a concentration of 10uM to reduce the release of exosome from DC. Co‐culturing for 8 hrs, the expression of adhesion molecules in HUVECs is determined. The results show that mature DCs can activate HUVECs and GW4869 can attenuate this effect. ***P < 0.001 (*n* = 3).

Dendritic cells are a group of immune cells which also perform as pro‐inflammatory cells by secreting cytokines. To clarify whether exosomes are involved in the process of endothelial inflammation induced by DCs, we co‐cultured BMDCs with HUVECs in a transwell system, which allows the transfer of both cytokines and exosomes but prevents the transfer of vesicles larger than exosomes and direct cell contact. GW4869, a well known inhibitor of exosome secretion, was used to reduce the release of exosomes from DCs 12. The results showed that mature DCs could activate HUVECs and GW4869 could attenuate this effect (Fig. 1). These data suggest that DCs contribute to endothelial inflammation and exosomes might be involved in the process.

### Successful culture of BMDCs with a non‐serum medium

To further confirm the role of exosomes in endothelial inflammation, we isolated exosomes from BMDCs culture medium (named DC‐exos) and then stimulated HUVECs with these DC‐exos. To rule out the effect of exosomes in foetal bovine serum, we used a non‐serum medium, X‐VIVO 15 (Lonza) 11, 14, to culture BMDCs. To test the validity of X‐VIVO 15 in culturing BMDC, BMDCs were directly observed through microscope at day 7 and the results showed a satisfied morphological structure (Fig. S1). Mature markers and inflammatory factors were also detected by flow cytometry, quantitative PCR and Elisa (Figs S2–S4). These data indicate a successful culture of BMDCs with X‐VIVO 15.

### Successful isolation of exosomes from DCs culture medium

**Figure 2:**
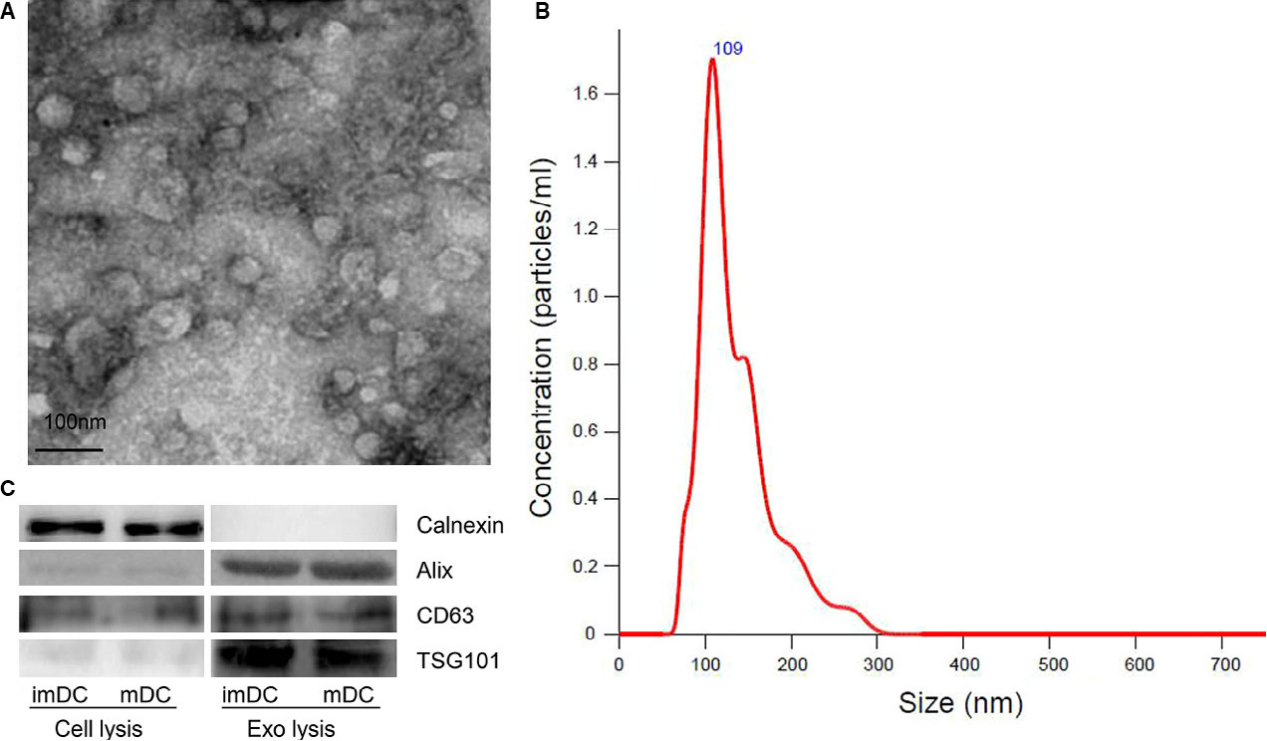
Successful isolation of exosomes from DCs culture medium. (**A**) The ultrastructure of mDC‐exos by transmission electron microscopy, bar size 100 nm. (**B**) The size distribution profile of mDC‐exos by Nanosight. (**C**) The expression of exosomes markers, CD63, Alix, TSG101 and negative marker Calnexin confirmed by immunoblotting. A total of 20 μg protein from DCs lysis and 5 μg protein from exosomes lysis was loaded into each lane (representative image of *n* = 3).

For exosomes isolating, BMDCs were treated as follows. At day 7 of DCs culture, PBS or LPS was added into the medium to generate immature or mature DCs. After 24 hrs of activation by PBS or LPS, the culture medium was replaced completely. Continuously culturing DC with another 48 hrs, exosomes were isolated from the culture medium using Exo‐Quick (System Bioscience). To adjust the interventional concentration of exosomes to HUVECs, we used 500 μl PBS to suspend exosomes isolated from 10 ml DC culture medium (10 × 10^6^ cells). The protein concentration was approximately 0.2 μg/ul, indicating about 100 μg exosomal protein extracted from 10 ml culture medium. As we previously reported, the total release, structures and sizes of exosomes from immature and mature DCs were similar 14. We observed the ultrastructure of exosome using transmission electron microscopy. Electron microscopic analysis revealed a typical size of 30–100 nm in diameter (Fig. 2A). To further investigate the size distribution profile of mature DC‐exos, we performed a size detection using the Nanosight, revealing a size peak of 109 nm (Fig. 2B). Then the expression of exosomes markers, Alix, CD63 and TSG101, were confirmed by immunoblotting (Fig. 2C). To test the purity of exosomes, we also determined the expression of Calnexin, which is a negative marker of exosomes 16. These data indicate a successful isolation of exosomes from culture medium.

### Isolated exosomes can induce inflammation of HUVECs through NF‐κB pathway

**Figure 3:**
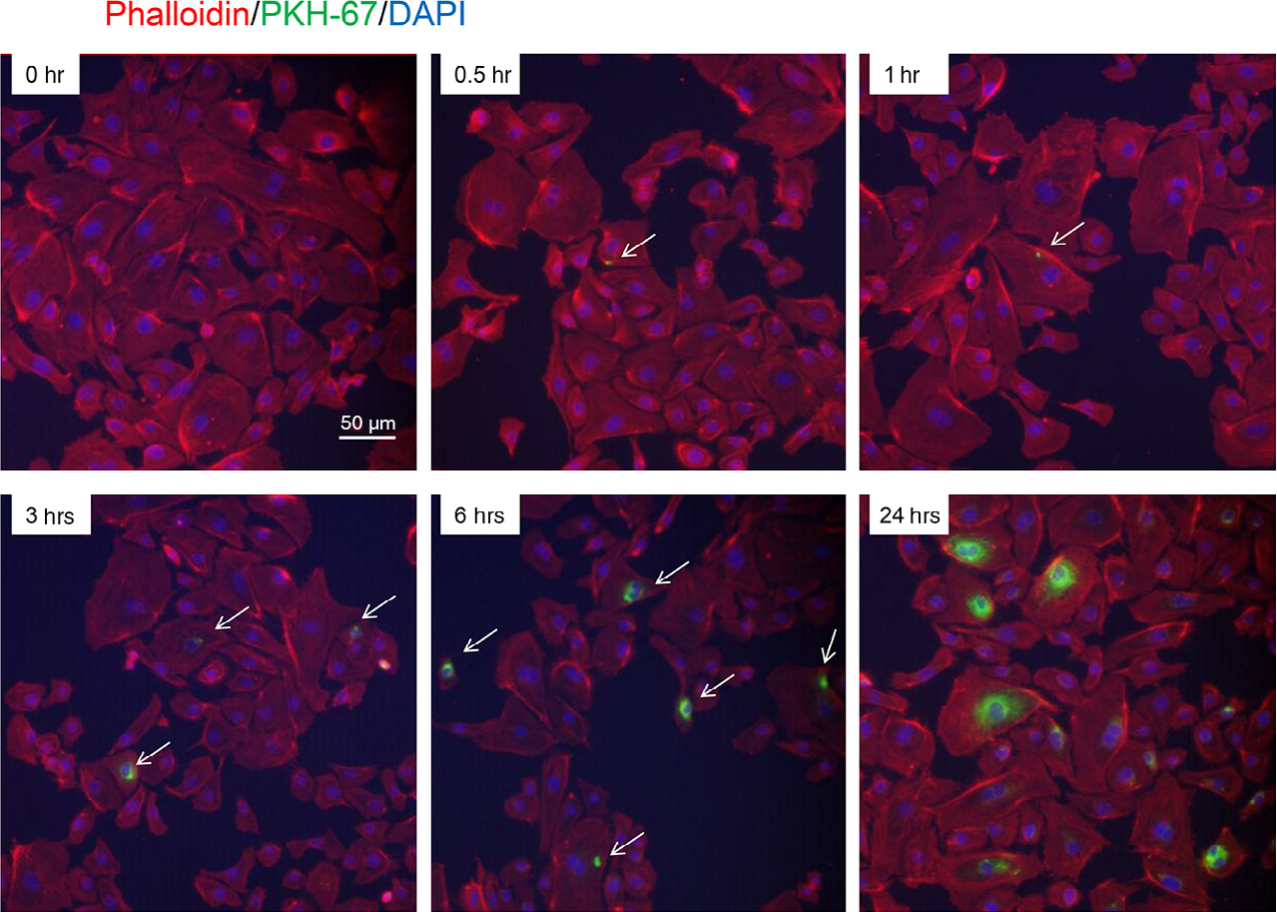
The uptake of exosomes by HUVECs. HUVECs were incubated with PKH67‐labelled mDC‐exos (green) and fixed for confocal imaging. HUVECs were stained with phalloidin (red) and nuclei with DAPI (blue). The incubated time was as indicated (representative image of *n* = 3).

**Figure 4:**
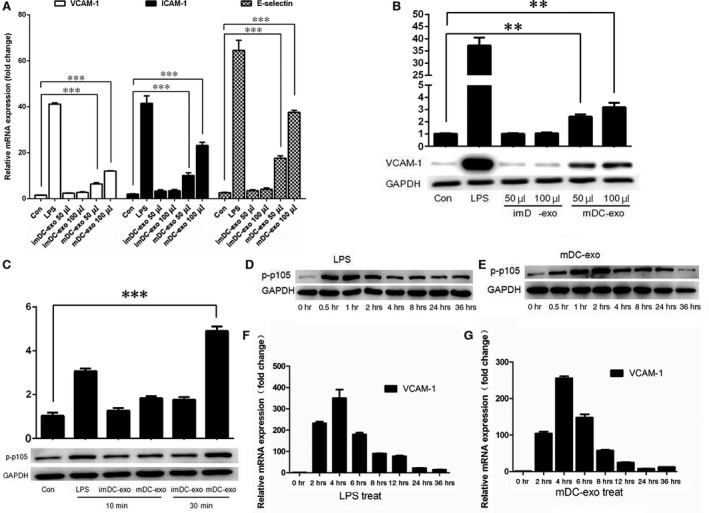
The activation of HUVECs induced by exosomes *via* NF‐κB pathway. (**A**) Exosomes from immature and mature DCs were incubated with HUVECs at indicated concentration for 8 hrs and the expression of adhesion molecules, VCAM‐1, ICAM‐1 and E‐Selectin, was detected by qPCR. (**B**) Exosomes from immature and mature DCs were incubated with HUVEC at indicated concentration for 24 hrs and the expression of VCAM‐1 was detected by immunoblotting. (**C**) Exosomes from immature and mature DCs were incubated with HUVEC for 10 or 30 min. and p‐p105 was detected by immunoblotting. (**D** and **E**) LPS or mDC‐exos were incubated with HUVECs for indicated time and p‐p105 was detected by immunoblotting. (**F** and **G**) LPS or mDC‐exos were incubated with HUVECs for indicated time and the VCAM‐1 expression was detected by qPCR. **P < 0.01, ***P < 0.001 (*n* = 3).

To study whether endothelial cells can uptake exosomes, we labelled DC‐exos with a green fluorescent marker, PKH67, and incubated them with HUVECs. The results showed that HUVECs could uptake exosomes as quick as 0.5 hr after incubation (Fig. 3). With prolonged incubation time, more exosomes were up‐taken by HUVECs. Then we further investigated the effect of DC‐exos on HUVECs. The expression of adhesion molecules was detected by qPCR and immunoblotting, which showed that mature DC‐exos could activate HUVECs in a dose‐dependent manner while immature DC‐exos had no such effect on HUVECs (Fig. 4A and B).

It has been reported that DC‐exos could affect target cancer cells by membrane TNF‐α 8 or inner contained miRNAs 9. Tumour necrosis factor‐α was well demonstrated to activate endothelial cells *via* NF‐κB pathway 17. Thus, we speculated that mature DC‐exos can stimulate HUVECs through membrane TNF‐α. To confirm this, we determined the phosphor‐p105 (p‐p105) expression in HUVECs activated by mature DC‐exos. We observed a rapid increase of p‐p105 induced by LPS (positive control, 10 min.) and mature DC‐exos (30 min.), indicating that mature DC‐exos could quickly activate HUVECs through NF‐κB pathway, possibly by the combination of exosome membrane protein and its receptor (Fig. 4C). To further understand the time manner of mature DC‐exos activation on HUVECs, we performed a time serial detection of p‐p105 and VCAM‐1 in HUVECs induced by LPS and mature DC‐exos. The results confirmed that mature DC‐exos activated HUVECs in a time manner similar to that of LPS, although the peak effect occurred at 0.5 hr for LPS and 2 hrs for mature DC‐exos (Fig. 4D–G). These data suggest that mature DC‐exos can stimulate HUVECs through NF‐κB pathway in a manner similar to that of LPS, indicating a rapid activation of HUVECs. The canonical pathway of activating NF‐κB signalling is through the ligation of TNF receptor 1 and soluble or membrane‐bound TNF‐α. According to the time manner and signal pathway of activation, we speculated a decisive role of exosomal TNF‐α in the process.

### TNF‐α in exosome membrane is involved in NF‐κB mediated injury of HUVECs

**Figure 5:**
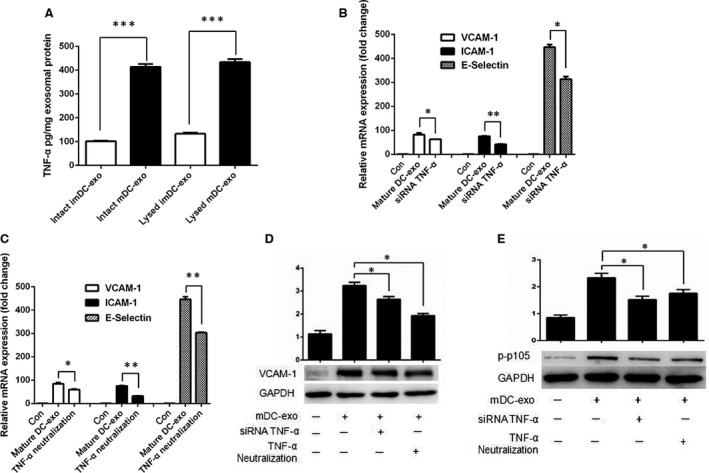
The NF‐κB pathway in HUVECs was activated by membrane TNF‐α in exosomes. (**A**) Untreated (Intact) or lysed (Lysed) immature DC‐exos and mature DC‐exos were examined by protein chip for the presence surface‐associated and total TNF‐α. (**B**) SiRNA TNF‐α were used to down‐regulate TNF‐α expression in DCs. Then the DC‐exos were isolated and treated to HUVECs for 6 hrs. Expression of VCAM‐1, ICAM‐1 and E‐Selectin was detected by qPCR. (**C**) Neutralizing antibody was used to block the TNF‐α bioactivities on exosomal membrane and expression of VCAM‐1, ICAM‐1 and E‐Selectin was detected by qPCR. (**D**) Exosomes treated with TNF‐α neutralizing antibody or from DCs treated with siRNA TNF‐α were incubated with HUVEC for 24 hrs and expression of VCAM‐1 was detected by immunoblotting. (**E**) Exosomes treated with TNF‐α neutralizing antibody or from DCs treated with siRNA TNF‐α were incubated with HUVEC for 30 min. and p‐p105 was detected by immunoblotting. *P < 0.05, **P < 0.01, ***P < 0.001 (*n* = 3).

Next, we performed a protein chip to examine protein expression in exosomes. To determine whether an interested protein was inside the exosome or on the membrane, exosomes were lysed or not before detection 8. If a protein is mainly located on the membrane, then the intact concentration should be as similar as the lysed concentration. Otherwise, the lysed concentration should be significantly higher than the intact concentration 8. Using this method, we found that TNF‐α was mainly expressed on the exosomal membrane and mature DC‐exos, compared with immature DC‐exos, displayed a higher expression of TNF‐α (Fig. 5A). We then used siRNA to knock down TNF‐α expression in DCs, with whose exosomes we activated HUVECs and found the expression of adhesion molecules decreased (Fig. 5B and D). To confirm this finding, we incubated neutralizing antibody with mature DC‐exos to block the TNF‐α bioactivities on exosomal membrane. We observed a decrease in adhesion molecules expression in HUVECs stimulated with mature DC‐exos (Fig. 5C and D). Finally, we determined the phosphor‐p105 (p‐p105) expression in HUVECs activated by mature DC‐exos which previously had been treated with siRNA TNF‐α or neutralizing antibody. The results showed a significant reduction in p‐p105 induced by siRNA TNF‐α and neutralizing antibody treated exosome (Fig. 5E). These data indicate that mature DC‐exos membrane express high level of TNF‐α, which can activate HUVECs directly and rapidly.

### Intravenous administration of mature DC‐exos induce endothelial inflammation and atherosclerosis in mice

**Figure 6:**
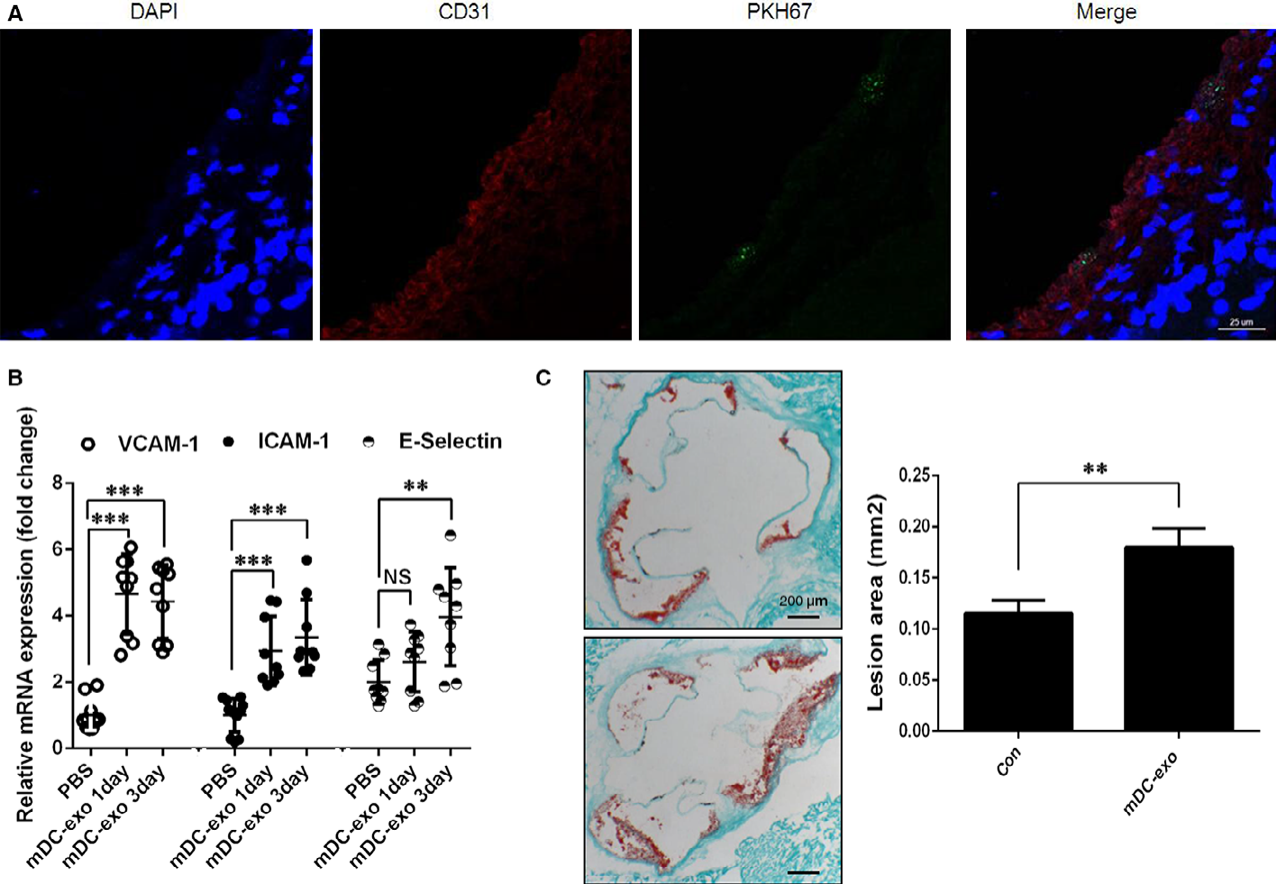
Exosomes from mature DCs induced endothelial inflammation and atherosclerosis *in vivo*. (**A**) PKH67‐labelled exosomes were intravenously injected to mice. After 24 hrs, aortas were carefully excised and stained with CD31 and DAPI. The images were acquired on an upright Leica SP8 confocal microscope. (**B**) Exosomes or PBS were injected to C57BL/6 mice. After 24 hrs or 3 days, the expression of VCAM‐1, ICAM‐1 and E‐Selectin in aortas was detected by qPCR (*n* = 9–11 mice per group). (**C**) ApoE−/− mice were fed a high fat diet and received weekly tail vein injections of mDC‐exos or PBS for 12 weeks as described in the Materials and methods section. Atherosclerotic Lesions in aortic sinus sections were stained with Oil Red O. Left, representative images in control and mDC‐exos groups. Right, comparison of lesion areas between control and mDC‐exos groups (*n* = 6 mice per group). **P < 0.01, ***P < 0.001, NS, non‐significant.

NF‐κB‐mediated endothelial cell activation and vascular inflammation plays a critical role in the initiation and progression of atherosclerosis 18. To study the *in vivo* effect of exosomes, we intravenously injected PKH67‐labelled exosomes into C57BL/6 mice. After 24 hrs, the aorta was excised and stained with CD31. We observed that these exosomes could be up‐taken by aortic endothelial cells (Fig. 6A). To explore whether mature DC‐exos is able to activate endothelial cell *in vivo*, we injected mature DC‐exos (20 μg in 100 μl PBS) into mice through tail vein. After 24 hrs or 3 days, the mice were killed and the aortas were detected for adhesion molecules expression. The results showed that the aorta endothelial was activated 24 hrs after a single injection hours and maintained to at least 3 days (Fig. 6B). Finally, we demonstrated that weekly injection of 20 μg mature DC‐exos for a period of 12 weeks could increase atherosclerotic lesions in ApoE−/− mice (Fig. 6C).

## Discussion

In this study, we demonstrate that DC‐exos are involved in endothelial inflammation through exosomal TNF‐α mediated NF‐κB pathway. Furthermore, *in vivo* study shows that the exosomes can be up‐taken by aortic endothelial cells and induce an inflammation and atherosclerosis. These findings highlight a new mechanism by which DC‐exos initiate endothelial injury and inflammation. Upon release from parent cells, exosomes distribute in body fluids and exert biological function remotely. This feature provides the possibility that DCs can influence endothelial cells without direct contact.

The relationship between DCs and atherosclerosis has been well reviewed 2, 3, 19. Dendritic cells pre‐exist under the endothelium in an immature state, even in arteries of young individuals still unaffected by any form of atherosclerosis. However, the majority of DCs in advanced plaques seems to be activated and they rapidly expand during atherosclerotic lesion growth. In the sub‐endothelial space of the aorta, DCs could efficiently accumulate lipids and therefore contribute to initiation and further progression of the disease. Meanwhile, DCs are capable of producing a number of pro‐inflammatory cytokines, including TNF, IL‐6 and IL‐12, which are pro‐atherogenic. This study expanded our knowledge, demonstrating the role of exosomes in the inflammatory progress.

Classically in cell biology, cells communicate with each other through direct interaction and/or by secreting soluble factors such as hormones, growth factors and cytokines. These soluble factors can act on the cell itself (autocrine signalling) or have an impact on both neighbouring (paracrine signalling) and distant cells (endocrine signalling). Interestingly, during the past decade, exosomes have become recognized as potent vehicles of intercellular communication. They have been isolated from most cell types and biological fluids such as saliva, urine, nasal and bronchial lavage fluid, amniotic fluid, breast milk, plasma, serum and seminal fluid 5. In this study, we showed that DCs can be a source of exosomes and these exosomes presented a functional role in inflammation and atherosclerosis.

Currently, we think exosomes are formed within the endosomal network and released upon fusion of multi‐vesicular bodies with the plasma membrane 5. Thus, in general, exosomes are highly abundant in cytoskeletal‐, cytosolic‐, heat shock‐ and plasma membrane proteins, as well as in proteins involved in vesicle trafficking. However, exosomes can also interact with target cells through a ligand‐to‐receptor interaction, including MHC I and II, transferrin receptors, tetraspanins and heat shock proteins (HSP) 5. Among ligand‐to‐receptor interactions, noteworthy are those recently identified, such as HSP70 through which plasma exosomes protect the myocardium from ischaemia‐reperfusion injury 20 and angiotensin II type I receptor through which cardiomyocyte derived exosomes modulate vascular responses to neurohormonal stimulation 21.

A widely investigated group of exosome cargos is miRNAs, which has attracted a huge attention in all research fields since the beginning of its discovery in exosomes. Exosomes and exosomal miRNAs have been well discussed in cardiac repair after myocardial infarction 6, 22, 23. Here, in this study, we observe that HUVECs are activated by mature DC‐exos in a pattern similar to that by LPS, indicating a rapid and direct interaction between DC‐exos and HUVECs, which is probably accomplished by ligand‐receptor response but not miRNAs. Thus, we performed a protein microarray to detect whether DC‐exos contain the major cytokines expressed by DCs, including TNF‐α. We found an elevated expression of TNF‐α in mature DC‐exos and they were mainly expressed on exosomal membrane. This finding was consistent with previous studies 8, 24. We then down‐regulated TNF‐α in DC‐exos and observed that the expression of adhesion molecules by HUVECs were attenuated, indicating a direct interaction between DC‐exos and HUVECs by TNF‐α.

It is well known that TNF‐α is among those pro‐inflammatory cytokines secreted by DCs upon stimulation. In this study we expanded our knowledge by illustrating that DCs can release TNF‐α contained exosomes which possess the function similar to soluble TNF‐α. Furthermore, exosomes uptake by target cells goes through membrane fusion, which requires a similar fluidity between the two fusing membranes, and both exosomes and plasma membranes display the same fluidity at pH 5.0 25, 26. It is conceivable, therefore, that when a functional molecule is delivered by exosomes, it may be more active than in its soluble form. One clear example of this is the ligands for death receptors, which are more functional when expressed on a membrane than in their soluble form 27, 28.

Since exosomes release can be systemically inhibited by GW4869, some studies have investigated whether the reduction of exosomes in serum can treat diseases. Indeed, they found that systemically administration of GW4869 could reduce the total exosomes in serum and treat allergic, cardiac and Alzheimer's dysfunction 29, 30, 31. Thus, our findings provide a potential method to prevent endothelial inflammation and atherosclerosis.

In summary, our study demonstrates that DCs are involved in the accelerated endothelial inflammation and atherosclerosis partly because of the released exosomes, which activate endothelial cells *via* TNF‐α mediated NF‐κB pathway. Our findings extend our knowledge on how DCs affect inflammation and provide a potential method to prevent endothelial inflammation and atherosclerosis.

## Conflict of interest

The authors confirm that there are no conflicts of interest.

## Supporting information


**Table S1** Table Primers for Real time qPCR.
**Figure S1** Morphological structure of BMDCs cultured with X‐VIVO 15 at day 7.
**Figure S2** Mature markers detected by flow cytometry in immature DCs and mature DCs cultured in X‐VIVO 15. *P<0.05.
**Figure S3** Inflammatory factors detected by quantitive PCR in immature DCs and mature DCs cultured in X‐VIVO 15. ***P<0.001.
**Figure S4** Inflammatory factors detected by Elisa in immature DCs and mature DCs cultured in X‐VIVO 15. ***P<0.001.
