# Supplementary material for: Exosomes derived from mature dendritic cells increase endothelial inflammation and atherosclerosis via membrane TNF‐α mediated NF‐κB pathway
Source: J Cell Mol Med. 2016 Aug 12;20(12):2318–27. doi: 10.1111/jcmm.12923 (PMC5134386; doi:10.1111/jcmm.12923)
Supplement: Supplementary file 1 — Table S1 Table Primers for Real time qPCR. Figure S1 Morphological structure of BMDCs cultured with X‐VIVO 15 at day 7. Figure S2 Mature markers detected by flow cytometry in immature DCs and mature DCs cultured in X‐VIVO 15. *P<0.05. Figure S3 Inflammatory factors detected by quantitive PCR in immature DCs and mature DCs cultured in X‐VIVO 15. ***P<0.001. Figure S4 Inflammatory factors detected by Elisa in immature DCs and mature DCs cultured in X‐VIVO 15. ***P<0.001. [file JCMM-20-2318-s001.doc]

Exosomes derived from mature dendritic cells increase endothelial inflammation and atherosclerosis via membrane TNF-α mediated NF-κB pathway

Supplemental Table, Figure and figure legend

Supplemental Table 1. Table Primers for Real time qPCR.

| name | Sequence (5' −> 3') |
| --- | --- |
| human VCAM‐1 forward | GCTGCTCAGATTGGAGACTCA |
| human VCAM‐1 reverse | CGCTCAGAGGGCTGTCTATC |
| human ICAM‐1 forward | TCTGTGTCCCCCTCAAAAGTC |
| human ICAM‐1 reverse | GGGGTCTCTATGCCCAACAA |
| human E‐selectin forward | AATCCAGCCAATGGGTTCG |
| human E‐selectin reverse | GCTCCCATTAGTTCAAATCCTTCT |
| human GAPDH forward | ATGGGGAAGGTGAAGGTCG |
| human GAPDH reverse | GGGGTCATTGATGGCAACAATA |
| mouse IL-1 forward | TCCAGGATGAGGACATGAGCAC |
| mouse IL-1 reverse | GAACGTCACACACCAGCAGGTTA |
| mouse IL-6 forward | CCACTTCACAAGTCGGAGGCTTA |
| mouse IL-6 reverse | GCAAGTGCATCATCGTTGTTCATAC |
| mouse TNF-α forward | GTTCTATGGCCCAGACCCTCAC |
| mouse TNF-α reverse | GGCACCACTAGTTGGTTGTCTTTG |
| mouse actin forward | GAAATCGTGCGTGACATCAAAG |
| mouse actin reverse | TGTAGTTTCATGGATGCCACAG |
| mouse VCAM-1 forward | GTTCCAGCGAGGGTCTACC |
| mouse VCAM-1 reverse | AACTCTTGGCAAACATTAGGTGT |
| mouse E-selectin forward | ATGCCTCGCGCTTTCTCTC |
| mouse E-selectin reverse | GTAGTCCCGCTGACAGTATGC |
| mouse ICAM-1 forward | GTGATGCTCAGGTATCCATCCA |
| mouse ICAM-1 reverse | CACAGTTCTCAAAGCACAGCG |


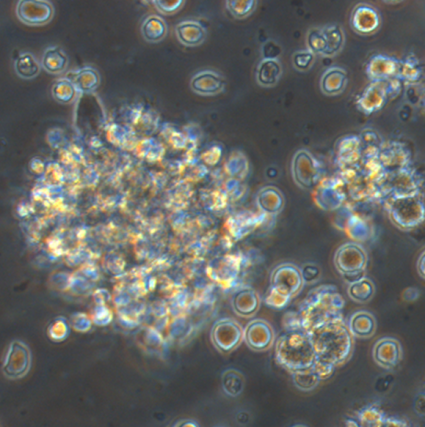


Supplemental Figure I. Morphological structure of BMDCs cultured with X-VIVO 15 at day 7.


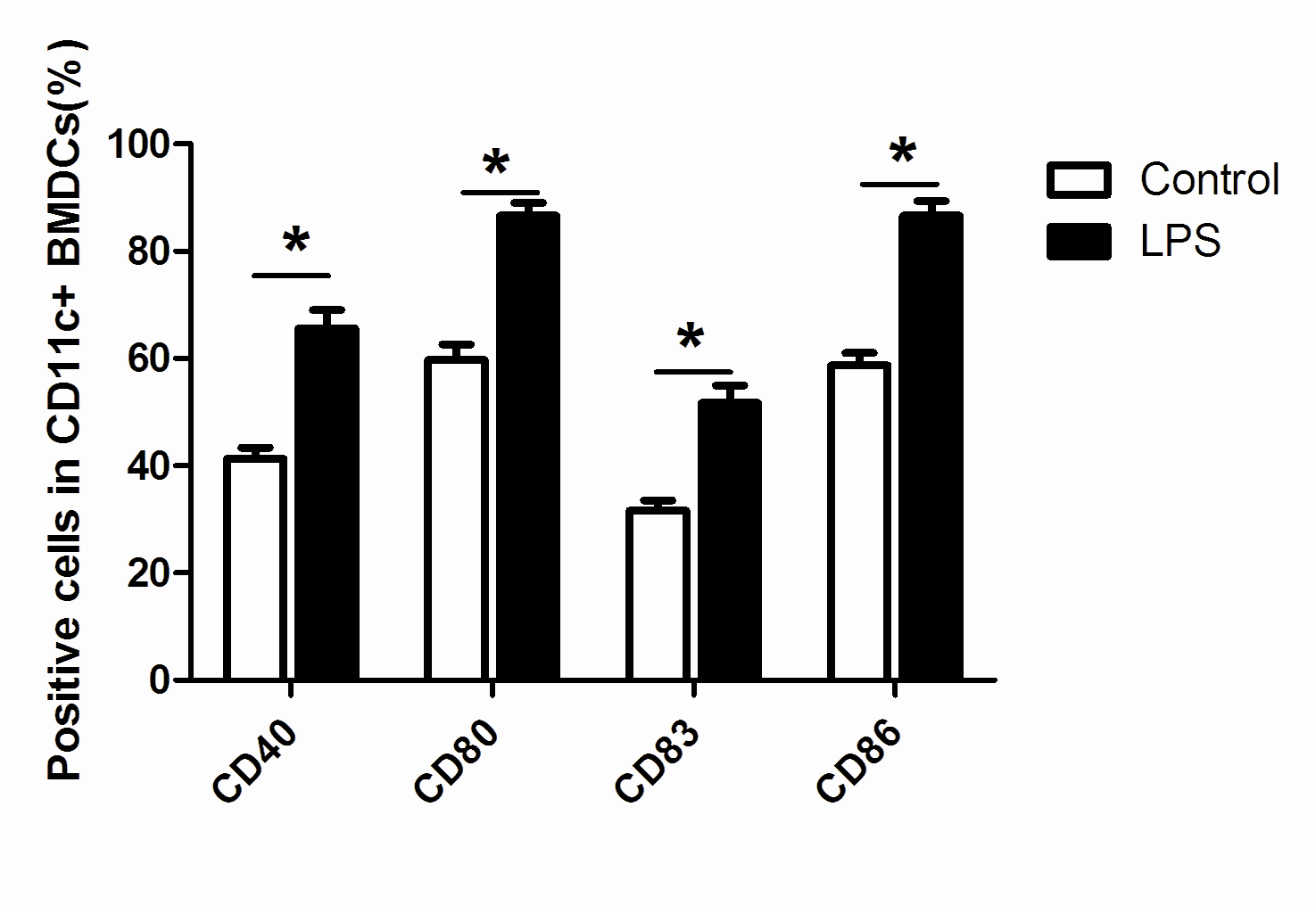


Supplemental Figure II. Mature markers detected by flow cytometry in immature DCs and mature DCs cultured in X-VIVO 15. *p<0.05.


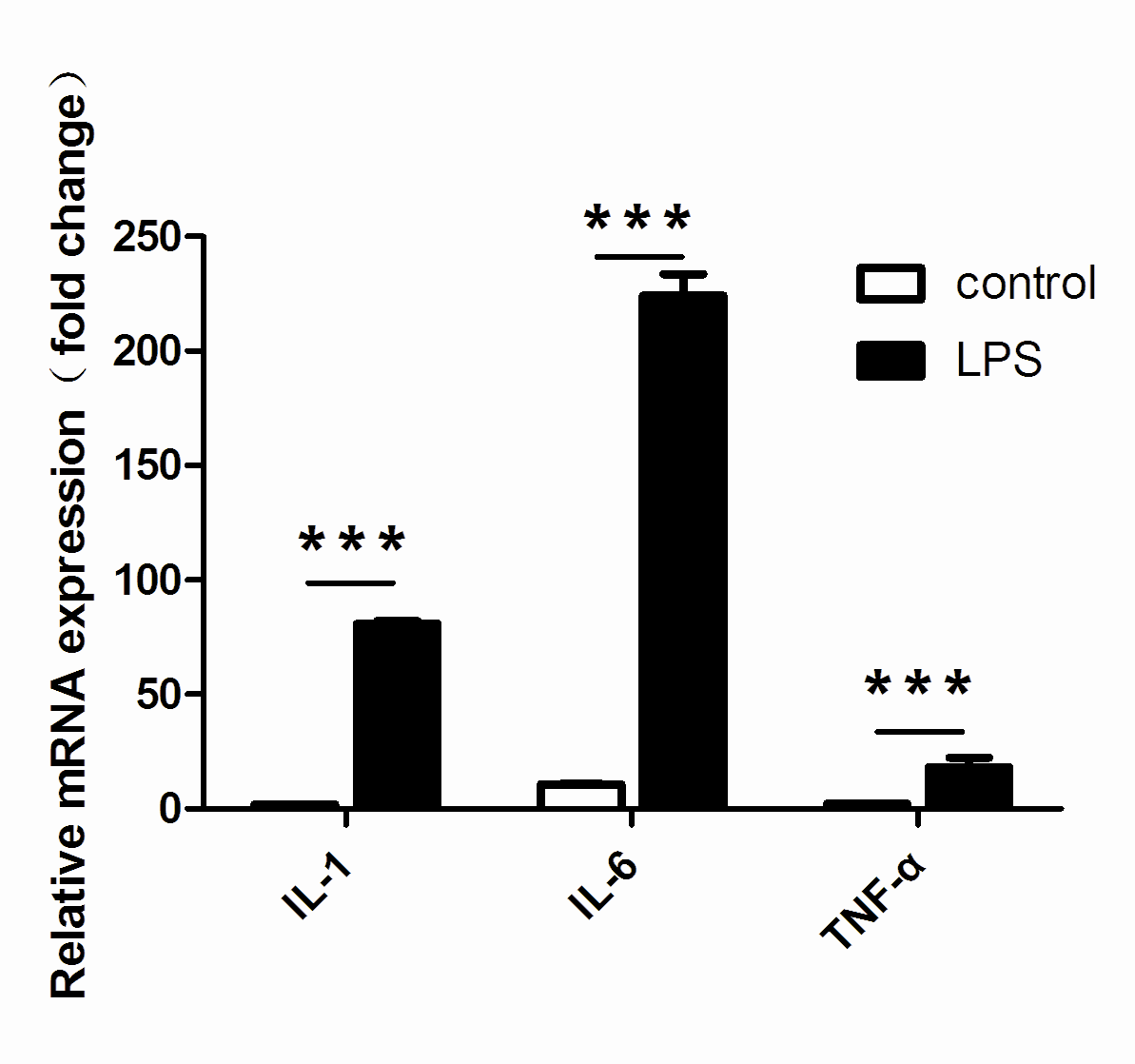


Supplemental Figure III. Inflammatory factors detected by quantitive PCR in immature DCs and mature DCs cultured in X-VIVO 15. ***p<0.001.


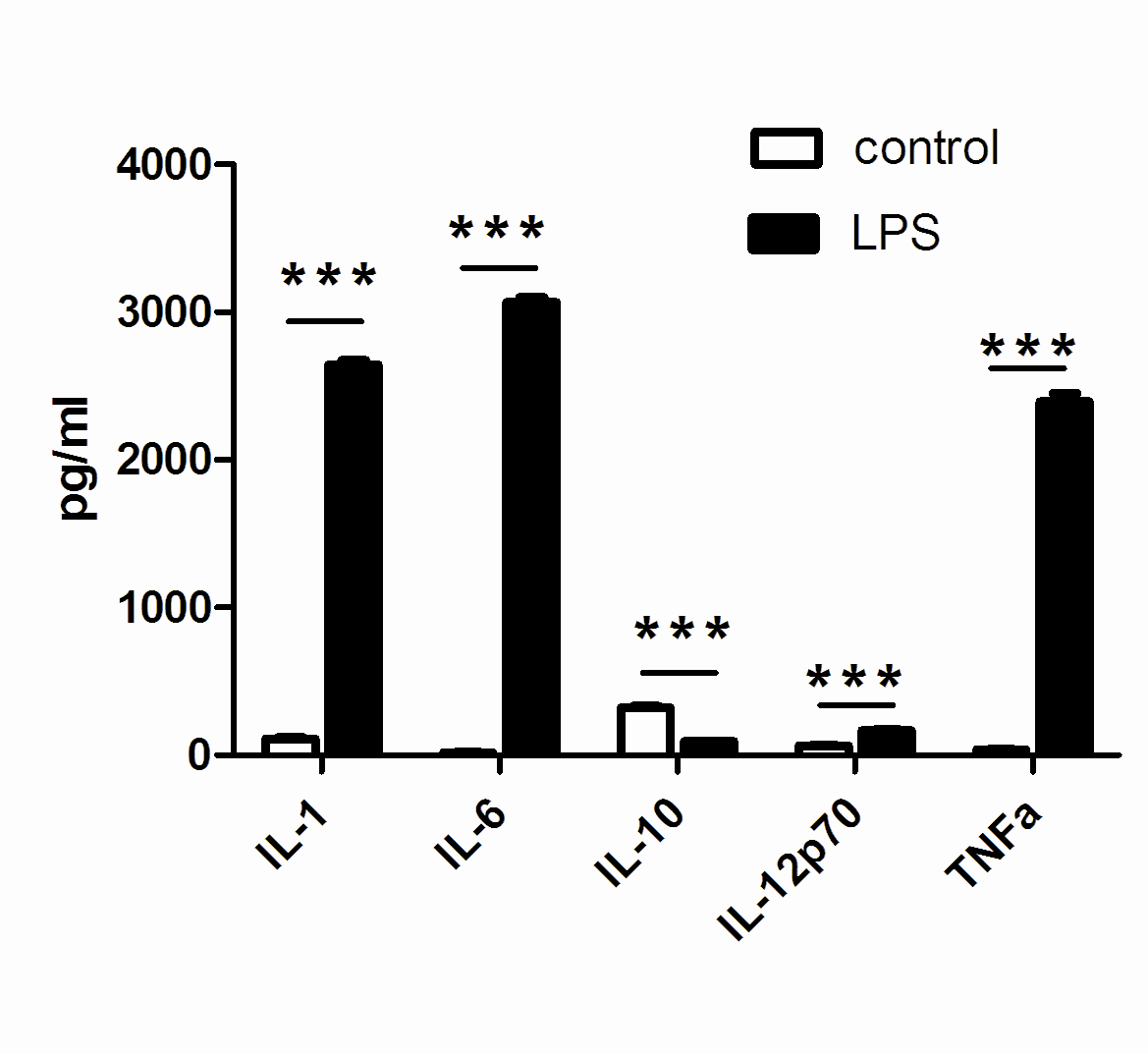


Supplemental Figure IV. Inflammatory factors detected by Elisa in immature DCs and mature DCs cultured in X-VIVO 15. ***p<0.001.
